# Current Genetic Survey and Potential Gene-Targeting Therapeutics for Neuromuscular Diseases

**DOI:** 10.3390/ijms21249589

**Published:** 2020-12-16

**Authors:** Wei Chiu, Ya-Hsin Hsun, Kao-Jung Chang, Aliaksandr A. Yarmishyn, Yu-Jer Hsiao, Yueh Chien, Chian-Shiu Chien, Chun Ma, Yi-Ping Yang, Ping-Hsing Tsai, Shih-Hwa Chiou, Ting-Yi Lin, Hao-Min Cheng

**Affiliations:** 1 Department of Medicine, National Yang-Ming University, Taipei 11221, Taiwan; alv320429@gmail.com (W.C.); michaelchang1109@gmail.com (K.-J.C.); yj1007hsiao@yahoo.com (Y.-J.H.); g39005005@gmail.com (Y.C.); d49624011@gm.ym.edu.tw (Y.-P.Y.); shchiou@vghtpe.gov.tw (S.-H.C.); 2 Department of Psychology, University of Toronto, Toronto, ON M1C 1A4, Canada; monica880325@gmail.com; 3 Department of Biological Science, University of Toronto, Toronto, ON M1C 1A4, Canada; 4 Institute of Clinical Medicine, National Yang-Ming University, Taipei 11221, Taiwan; 5 Division of Basic Research, Department of Medical Research, Taipei Veterans General Hospital, Taipei 112201, Taiwan; yarmishyn@gmail.com (A.A.Y.); figatsai@gmail.com (P.-H.T.); 6 Department of Medical Research and Education, Taipei Veterans General Hospital, Taipei 112201, Taiwan; polo661124@yahoo.com.tw; 7 Institute of Pharmacology, National Yang-Ming University, Taipei 11221, Taiwan; 8 Department of Medicine, National Taiwan University, Taipei 10617, Taiwan; joseph7758758@gmail.com; 9 Institute of Food Safety and Health Risk Assessment, National Yang-Ming University, Taipei 11221, Taiwan; 10 Genomic Research Center, Academia Sinica, Taipei 11529, Taiwan; 11 Center for Intelligent Drug Systems and Smart Bio-devices (IDS2B), National Chiao-Tung University, Hsinchu 1001, Taiwan; 12 Department of Medicine, Kaohsiung Medical University, Kaohsiung 80708, Taiwan; 13 Center for Evidence-based Medicine, Taipei Veterans General Hospital, Taipei 112201, Taiwan

**Keywords:** gene therapy, CRISPR, neuromuscular disease, duchenne muscular dystrophy, spinal muscular atrophy

## Abstract

Neuromuscular diseases (NMDs) belong to a class of functional impairments that cause dysfunctions of the motor neuron-muscle functional axis components. Inherited monogenic neuromuscular disorders encompass both muscular dystrophies and motor neuron diseases. Understanding of their causative genetic defects and pathological genetic mechanisms has led to the unprecedented clinical translation of genetic therapies. Challenged by a broad range of gene defect types, researchers have developed different approaches to tackle mutations by hijacking the cellular gene expression machinery to minimize the mutational damage and produce the functional target proteins. Such manipulations may be directed to any point of the gene expression axis, such as classical gene augmentation, modulating premature termination codon ribosomal bypass, splicing modification of pre-mRNA, etc. With the soar of the CRISPR-based gene editing systems, researchers now gravitate toward genome surgery in tackling NMDs by directly correcting the mutational defects at the genome level and expanding the scope of targetable NMDs. In this article, we will review the current development of gene therapy and focus on NMDs that are available in published reports, including Duchenne Muscular Dystrophy (DMD), Becker muscular dystrophy (BMD), X-linked myotubular myopathy (XLMTM), Spinal Muscular Atrophy (SMA), and Limb-girdle muscular dystrophy Type 2C (LGMD2C).

## 1. Introduction

**Figure 1:**
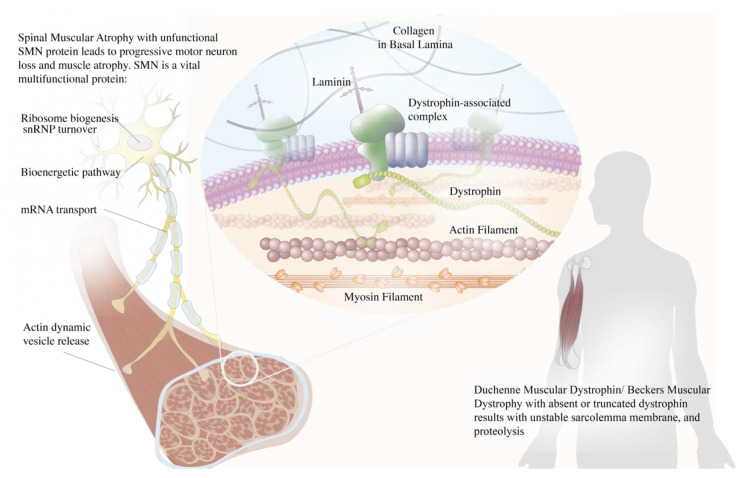
Schematic illustration of the roles of dystrophin and survival motor neuron (SMN) in neuromuscular diseases. The dystrophin acts as a muscle anchor protein, connecting the actin cytoskeleton to the dystrophin–glycoprotein complex and stabilizing the sarcolemma. Dysfunctional dystrophin leads to an unstable sarcolemma membrane and muscle proteolysis. The SMN plays numerous roles in a distinct compartment of the motor neuron that is crucial for its survival, encompassing ribosomal biogenesis, ubiquitin homeostasis, Cajal body turnover, mRNA transport, and actin dynamic vesicle release. Dysfunctional SMN leads to motor neuron death and subsequent muscle atrophy.

Neuromuscular diseases (NMDs) are a disease entity characterized by functional impairment of the motor neuron–muscle functional axis components (Figure 1). Inherited monogenic neuromuscular disorders encompass both muscular dystrophies and motor neuron diseases. Apart from the classic gene augmentation of delivering functional genes, researchers have devised various approaches to exploit the gene expression machinery to bypass splicing aberrations or premature stop codons (PTCs) to achieve therapeutic protein expression. NMDs present with a myriad of intractable diseases that through the advent of next-generation sequencing and genetic tools are beginning to witness the unprecedented clinical translation of gene therapies. Researchers are devising fascinating gene targeting methods that target the style of mutation and also strategic vector delivery that circumvents traditional limitations. We delineate for each disease the pathophysiology, current clinical trial status and limitations, and ongoing preclinical studies that are expected to surge. As NMDs are caused by vast yet underexplored mutations, researchers aim to de-personalize gene therapy in the hope of broadening the targetable patients with one drug. Not only does CRISPR/Cas9 offer a permanent cure of disease by correcting the genetic defect at its roots, CRISPR are also used to target previously neglected mutations. In this review article, we focused on the current development of genome editing on NMDs that are available in published reports, including Duchenne Muscular Dystrophy (DMD), Becker muscular dystrophy (BMD), X-linked myotubular myopathy (XLMTM), Spinal Muscular Atrophy (SMA), Limb-girdle muscular dystrophy Type 2C (LGMD2C) and Fascioscapulohumeral Muscular Dystrophy (FSHD).

## 2. Duchenne Muscular Dystrophy (DMD) and Becker Muscular Dystrophy (BMD)

DMD and BMD are X-linked recessive diseases caused by the mutations in the *DMD* gene (Xp21) [1] and the consequent loss of dystrophin protein. The *DMD* gene is one of the largest human genes with the size of 2.4 Mb and being composed of 79 exons that encode 14 kb of cDNA [2]. Since such large cDNA is beyond the carrying capacity of the viral vectors (5 kb), researchers attempted to deliver shorter versions of the *DMD* gene for gene therapeutic purposes, such as micro-dystrophin (3.7–4.9 kb) and mini-dystrophin genes (6.3 kb) [3]. Such transcripts are translated into shorter micro-dystrophin (150–299 kDa) and mini-dystrophin (427 kDa) proteins that still preserve the function of operating in the Dystrophin Glycoprotein Complex (DGC) [4]. The dystrophin protein connects the actin cytoskeleton to the DGC, anchoring the muscle extracellular matrix and stabilizing sarcolemma in muscle contraction. Thus, dystrophin’s absence induces muscle degradation that typically onsets around the age of two to five with symptoms such as gait abnormalities, positive Gower’s sign, and progressive deterioration in proximal muscle strength [5,6].

**Figure 2:**
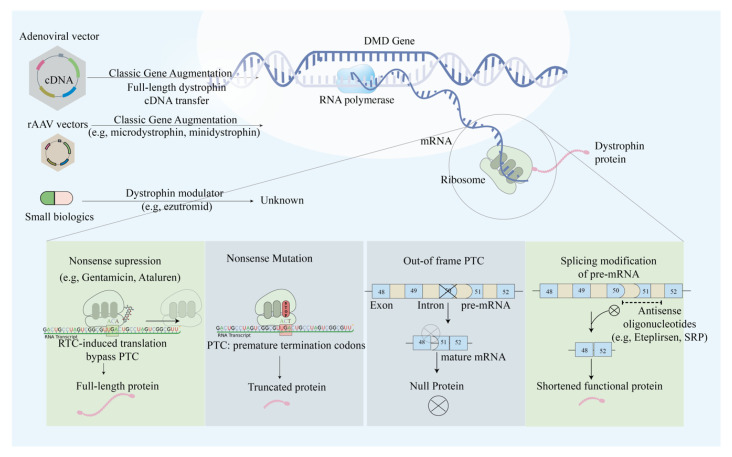
Schematic illustration of the main approaches in modulating dystrophin expression via genetic manipulations. The main approaches include nonsense suppression that allows PTC readthrough, exon skipping to restore reading frame, classic gene augmentation of full, micro- and mini-dystrophin, and genetic modulators that augment mRNA expression.

Previously, apart from the administration of anti-inflammatory agent prednisone that delays muscle proteolysis [7], no alternative treatment existed for DMD patients. Researchers recently targeted different mutation types with different gene therapy approaches, ranging from exon skipping, readthrough of stop codons, gene transfer, pseudo exon activation, and alternative splicing (Figure 2). Exon skipping therapy aimed to restore the protein expression in out-of-frame and in-frame deletions in the *DMD* gene [8]. Out-of-frame deletions result in truncated or null protein products, which leads to life-threatening phenotype. On the contrary, in-frame deletion in which the mutation does not disrupt the whole translational reading frame leads to a milder phenotype referred as BMD [9]. Researchers devised antisense oligonucleotides (AOs) to induce exon skipping at the pre-mRNA level [10].

### 2.1. Single Exon Skipping

Antisense oligonucleotides (AOs) are short nucleic acid sequences designed to splice the *DMD* gene in a way to skip an exon with the mutation that disrupts the open reading frame, thus resulting in BMD-like functional dystrophin. The sequences selectively bind to pre-mRNA, forming small double-stranded regions at the key sites where spliceosomes bind. The mutated exons are skipped, and the reading frame is restored, albeit in a shorter form [11,12]. To restore the translational reading frame, researchers cut off a single or multiple exons that contain premature stop codons. In two studies, myotube cultures extracted from DMD patients with exon 45 deletion were treated with AO-induced exon 46 skipping therapy and showed that up to 80% of dystrophin was restored [8,10]. The same therapeutic strategy was later applied to cultured muscle cells derived from six DMD patients, each with different exon deletions and point mutations [10].

In each case, dystrophin synthesis could be restored by 75% in myotube cells. These two studies serve as the cornerstone for antisense-based therapy for DMD [10,13]. In the study of phosphorodiamidate morpholino oligomer (PMO)-induced exon 23 skipping delivered to the anterior tibialis muscle of mdx mouse, the percentage of dystrophin-positive myofibers significantly correlated with resistance to mechanical stress, and a minimum of 20% of the dystrophin-positive myofibers level was shown to be required to restore function. However, because of the regenerative capacity of mdx muscle, which is supported by only a 20–30% reduction of specific force compared to wild type, no significant correlation between dystrophin-positive myofibers and improvements in muscle force was observed. This provides a notion that an evaluation of functional changes associated with dystrophin restoration might not be a sensitive indicator of the real effects [14].

Among published clinical trials (Table 1 and Table 2), Drisapersen (PRO051 or GSK2402968), a 2′O methyl phosphorothioate (2′OMePS) AO developed by Prosensa Holding N.V., and Eteplirsen (AVI-4658), a PMO developed by Sarepta Therapeutics, Inc. underwent intensive evaluation. In 2007, researchers demonstrated safe and competent intramuscular Eteplirsen delivery to induce local dystrophin expression on ambulant boys that possessed deletion in exon 51 in a dose escalation, proof of concept trial (NCT00159250) [15]. Two years later, in phase 2, a dose-escalation trial in DMD patients under the selection of amenable deletions achieved no drug-related adverse events while achieving a significant observation of dose-dependent dystrophin expression (NCT00844597) [16]. 

As for Drisapersen, the proof-of-principle study recruited four patients eligible for exon 51 skipping correction that received Drisapersen intramuscular injection and showed efficient dystrophin restoration [17]. In 2010, a randomized phase 2 trial was launched, and researchers found that early-stage young DMD patients receiving the drug improved in ambulation (NCT01153932). Nevertheless, conflicting trial evidence suggests that perhaps the drug will only benefit a subpopulation of patients that suffers with less severity. A 48-week, randomized placebo-controlled phase 3 trial enrolled 186 boys with *DMD* gene exon 51 skipping and aimed to evaluate the efficacy and safety of Drisapersen (NCT01254019). No significant improvements of efficacy endpoints (6MWD minute-walking distance, 4-stair climb ascent velocity, and 10-m walk/run velocity) over the population were shown after 48 weeks. The uneven distribution of age and baseline performance in Drisapersen and placebo groups (Drisapersen group with higher age and higher disease severity) increased data variation compared to the former phase 2 study. They suggest that the low efficacy of gene therapy on more impaired patients (baseline 6MWD <300 m) might be a contributing factor to the unexpected result [18]. Despite advances of exon-skipping drugs, researchers are exploring possible solutions to the limitations of such an approach to target the multitude of transcripts generated in DMD. The *DMD* gene comprises 79 exons and can generate many potential transcripts, making AOs’ design a challenge. Secondly, the truncated dystrophin products produced by different AO designs vary in functionality. Finally, although the DMD phenotype becomes milder, the technology does not correct the underlying dystrophin mutation. 

### 2.2. Multi-Exon Skipping 

Applicability of single exon skipping is limited because mutation patterns vary in each case, raising concerns about whether splicing on a single site benefits patients [9,19]. To expand the coverage of patients benefiting from a single drug, researchers gravitated toward devising multi-exon skipping or AO cocktail sets. The deletion of repeats 4 to 23 (DeltaR4-R23) of the dystrophin-coding sequence can still generate functional dystrophin and theoretically can be applied to patients with mutations within the mutation range of DeltaR4-R23 [20]. Therefore, it supports the feasibility of deleting multiple consecutive exons and produce functional protein. Analyzing out-of-frame deletion mutations, researchers identified exons 45–55 as the dystrophin gene mutation hotspot, accounting for almost 50% of DMD patients [21,22,23]. Several AO cocktails achieved skipping of up to 10 exons in vitro and in vivo [24]. The tailored cocktail combinations offer higher hopes in expanding patients’ coverage with out-of-frame deletions than single exon skipping and enhancing motor dysfunction alleviation. Nevertheless, safety drug approval regulations demand that each cocktail component pass the safety test hurdle [25], since the AO approach would induce more than one exon to be skipped.

### 2.3. Mutation Suppression by Readthrough of Stop Codons

In one of eight DMD patients [26], the nonsense mutations occur when the premature termination codons (PTCs) are located in the coding regions of the *DMD* gene. The translation process is forced to terminate, resulting in truncated dysfunctional dystrophin protein forms. The readthrough of PTCs caused by several ribosome-binding compounds, including gentamicin (aminoglycoside) and Ataluren (PTC124), has been observed to lead to the restoration of full-length functional proteins [26,27]. As was first observed in 1999, aminoglycoside treatment could suppress the stop codon in cultured mammalian cells and mdx mice. Exposure of mdx myotubes to gentamicin resulted in restoration of the dystrophin protein, and the intravenous administration of gentamicin exhibited functional protection against injury. Despite the evidence of phenotypic suppression of PTCs, researchers raise concerns about its clinical implementations due to the need for high therapeutic concentration with a narrow window of functional dosage [28]. In 2007, Ataluren (Tranlarna, PTC124) was identified in a high-throughput small-molecule screen to bind to the ribosome and suppress nonsense mutations by promoting PTC readthrough. Ataluren has distinct advantages with its oral route delivery, high potency, and safe toxicity profile [29]. A proof-of-concept phase 2a trial showed the correlation of Ataluren and dystrophin restoration in muscle cells and Ataluren’s well-toleration by DMD patients [30,31,32]. Nevertheless, Ataluren delivery in two randomized controlled trials did not significantly improve strength, but the disease progression was delayed [31,32,33]. As DMD pathophysiology is only delayed and not reversed, discussions about Ataluren efficacy on DMD patients persists, and the mechanism of Ataluren function has not been clarified [34].

### 2.4. AAV-Based Mini- and Micro-Dystrophin Gene Transfer

Gene therapy in DMD aims to deliver mini- and micro-dystrophin, or alternative genetic materials into the target muscles. Interest in AAV-based gene transfer in DMD is fueled by recognizing that AAV’s multiple serotypes possess a natural tropism for muscle and allows for persistent and episomal gene expression. Nevertheless, such technology is challenged by the low cloning capacity of less than 5 kb. The full length of dystrophin cDNA is 14 kb; therefore, researchers addressed such limitations by devising micro-dystrophin and mini-dystrophin genes. They successfully ameliorated the muscular dystrophy phenotype in mdx mice using the truncated therapeutic proteins [3,35,36]. As mini-dystrophin cDNA has a length of 6–8 kb and cannot fit in a single AAV vector, researchers are challenged with identifying the smallest dystrophin gene that may still deliver the therapeutic effects. Micro-dystrophin, weighing 3.5–4 kb, satisfies functional dystrophin’s minimum requirements and has a size that fits into the viral vectors [3,37].

Preclinical data in animal models and previous clinical trials suggested that systemic AAV mini- or micro-dystrophin may be a viable treatment for DMD patients [38,39,40,41,42,43,44,45]. In March 2020, a phase 1/2a nonrandomized trial was launched by Nation Children’s Hospital, where 4 DMD patients received a single dose of SRP-9001(rAAVrh74.MHCK7.micro-dystrophin) delivered by limb IV therapy. The trial was concluded with a safe expression profile and both the expression of micro-dystrophin at the correct location and improvements in North Star Ambulatory Assessment (NSAA) (NCT03375164) [46]. Different teams used different AAV serotypes, expression cassette promoters, and the alternative gene for full-length dystrophin. Sarepta Therapeutics Inc. is responsible for trial SRP-9001, which was developed initially by Abigail Wexner Research Institute at Nationwide Children’s Hospital; Pfizer is responsible for trial PF-06939926 (AAV9-carried mini-dystrophin under control of muscle creatine kinase promoter); and Solid Biosciences is responsible for trial SGF-001 (AAV9 micro-dystrophin under control of CK8 promoter). It is worth noting that while most trials enrolled ambulatory DMD patients, Pfizer recruited non-ambulatory as well (NCT03769116, NCT03362502, NCT03368742).

In addition to the ongoing clinical trials, rigorous preclinical studies are still essentially needed to optimize the efficacy of AAV gene transfer. The coverage of more functional domains in micro- or mini-dystrophin is expected to result in better therapeutic outcomes. Dual AAV technology was demonstrated in the DMD canine model using 7 kb canine ΔH2–R15 mini-dystrophin. The dual AAV complex was cloned in Y731F tyrosine-modified AAV9 and delivered locally in a canine muscle. After 2 months of the injection, widespread mini-dystrophin expression was observed with dystrophin-associated glycoprotein complex restoration and functional protection from eccentric contraction-induced force loss [47]. Cytotoxic lymphocyte (CTL) response to mini-dystrophin products poses a risk to eliminate all the transduced muscle. An immunosuppression strategy, in which gene-encoding viral small peptide ICP47 is fused into transgenes, inhibits mini-dystrophin-specific CTL response [48]. An AAV9 micro-dystrophin vector can be generated in the herpesvirus system and have comparable biopotency to that made by the transient transfection method in the mouse model [49].

### 2.5. Anti-Myostatin Therapy

In contrast to delivering traditional augmentation therapies, researchers also explored the feasibility of inhibiting the skeletal muscle’s negative regulator, myostatin. Promising evidence of myostatin blockade or attenuation to improve muscle functions is demonstrated in early preclinical studies [50,51,52]. Anti-myostatin molecules including MYO-029 (a neutralizing antibody to myostatin), ACE-031 (a compartment of activin receptor type IIB and IgG1-Fc that binds myostatin and related ligands), and FS344 (modified follistatin, which is a potent myostatin antagonist) were developed and applied clinically to DMD or BMD patients. However, the results of clinical trials studying MYO-029 and ACE-031 were disappointing and showed no improvements in physical function [53,54]. Only Follistatin344 showed efficacy on BMD patients. A proof-of-concept phase 1/2a clinical trial was carried out on six BMD subjects, who were treated with AAV1.CMV.FS344 vector that exhibited a safe profile, and four of them made improvements in the 6MWD test [55].

The poor efficacy of the anti-myostatin strategy is discussed by Mariotet et al. Myostatin expression is highly reduced in atrophying DMD patients at both mRNA and serum protein levels. Only 8% of mRNA and 50.6%  ±  17.1% of circulating myostatin levels were detected as compared to the control group. The intrinsic down-regulation of myostatin in atrophying DMD is speculated to counterbalance the muscle wasting progress, and this may explain the contradictory results in preclinical studies and human trials [56]. Evidence of the down-regulation of myostatin was also found in a dystrophin-deficient Golden Retriever muscular dystrophy (GRMD) dog model [57]. To counter the challenges of anti-myostatin therapy, associated findings shed light on the augmentation of anti-myostatin and other muscle restoration therapies. A micro-dystrophin/follistatin combinatorial therapy completely restored resistance to eccentric contraction-induced injury in mdx mouse model [58]. In a study by Mariot et.al, myotubularin (Mtm1)-rescued Mtm1-KO mice were injected with an AAV-coded myostatin pro-peptide D76A mutant (AAV-PropD76A), and results were encouraging that muscle mass increased by 179%  ±  25.2% (*p * =  7.15) [56]. These two studies indicate that myostatin allows the restoration of muscle mass or function with partially rescued myofibers. On the other hand, in light of the promising results seen in BMD, a clinical intramuscular gene transfer to DMD patients trial just ended on April 15, 2020, and simultaneously, descriptive results can be found on ClinicalTrials.gov (NCT02354781)). Still, the results are yet to be published.

## 3. Spinal Muscular Atrophy (SMA)

**Figure 3:**
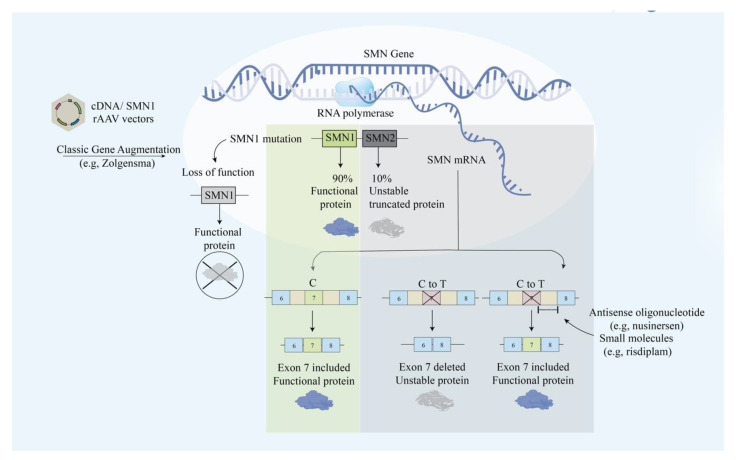
Illustrative scheme for the main approaches in modulating the SMN protein. The main approaches include exon skipping with antisense oligonucleotide and classic gene augmentation of SMN1.

SMA is an autosomal recessive neurodegenerative disease that causes progressive motor neuron loss in the central nervous system, peripheral nervous system, and skeletal muscles. The deletion of the *SMN1* gene causes the deficiency of SMN, which is a 38-kDa motor neuron protein that is crucial for survival (Figure 1) [59]. Most patients with SMA have abnormal *SMN1* with normal *SMN2* paralog. Although the nucleotide sequences of *SMN1* and *SMN2* are 99% identical, the loss of *SMN2* with intact *SMN1* has no clinical consequences. *SMN2* only translates into 10% of the functional SMN protein via an mRNA that includes exon 7 [60,61]. The distinction between the two genes lies within the coding region where a single nucleotide transition C-to-T at a codon 270 of exon 7 in *SMN2* results in an alternative splicing pattern [60]. The C-to-T mutation attenuates the exonic enhancer’s activity and suppresses the ability of *SMN2* to compensate for a dysfunctional *SMN1* [60]. Unfortunately, SMA has no cure yet and is mainly treated by intensive supportive care. Gene therapy approaches aim to restore *SMN1* translation and increase the SMN protein level (Figure 3).

### 3.1. Modification of SMN2 Alternative Splicing

Unraveling the molecular difference between *SMN1* and *SMN2*, researchers aimed to promote *SMN2* exon 7 expression to increase the level of full-length and functional SMN proteins. In vivo studies have supported this idea by successfully demonstrating the enhanced survival rate and increased body weight by administering small molecule enhancers of *SMN2* exon 7 inclusion in mice [62]. First, FDA approved treating SMA with nusinersen, which is an AO drug that increases full-length SMN translation by modifying pre-mRNA splicing of the *SMN2* gene. By blocking the intronic splicing silencer N1 (ISS-N1) downstream of exon 7, nusinersen corrects *SMN2* exon 7 splicing defect [63]. Miraculously, nusinersen achieved treatment response in 40% of patients, who reported improved motor movements such as head control, sitting, ability to kick in the supine position, rolling, crawling, standing, and walking [64], which are responses that were not observed in the control group. Nusinersen cannot cross the blood–brain barrier, therefore, it requires multiple intrathecal injections [65]. In addition to the administration method, there are also other limitations of nusinersen that need to be considered, including the side effects and the high price. A single dose of intrathecal injection Nusinersen (Spinraza) costs 118,000 dollars and there is an annual drug cost of 708,000 for the first year and 354,000 for subsequent years [57]. Patients reported adverse events such as back pain and post-lumbar-puncture headache after the treatment [66].

### 3.2. AAV-SMN

Zolgensma (onasemnogene abeparvovec) is an AAV9-based gene therapy approved in May 2019 that improves motor function and SMA patients’ survival rate [67]. Monani et al. showed that increasing the copy number rescued the SMA phenotype in mice, indicating that phenotypic severity can be modulated by direct augmentation of the *SMN2* gene. Zolgensma delivers a fully functional copy of the human *SMN1* gene under the control of cytomegalovirus enhancer/chicken-β-actin-hybrid promoter into the target motor neuron cells to increase levels of the functional SMN protein [31,68]. A phase 3 clinical study with one-time intravenous administration of Zolgensma with AAV9 vector containing cDNA of human *SMN1* gene under the control of the cytomegalovirus enhancer/chicken-β-actin-hybrid promoter has been tested with 59% achieved primary outcome of independent sitting for at least 30 s, and 90% achieved primary outcome of event-free survival (NCT03306277) (Table 3). Although the FDA has approved Zolgensma, the drug’s long-term effect is still unknown. There is currently a long-term safety follow-up study being conducted starting from 2018 and estimated to be completed in 2033. The study’s primary outcome is to collect long-term safety data from patients in the AVXS-101-CL-101 gene replacement therapy clinical trial for SMA Type 1 delivering Zolgensma for up to 15 years with annual follow-up visits (NCT03421977). Zolgensma is given as a single dose intravenous injection, which resolves the disadvantage of repeated intrathecal injection of nusinersen.

Attempting to circumvent the need for injections, Risdiplam and Branaplam were designed to be administered orally. Branaplam is a small molecule that corrects the splicing defect of *SMN2* and stabilizes the interaction between the spliceosome (U1 snRNP) and *SMN2* pre-mRNA [69]. The first clinical trial of Branaplam (LMI070) with a phase I/II was initiated in 2014 by Novartis (NCT02268552), but it was paused in 2016 after the canine experiment follow-up demonstrated long-term toxicity and axonal degeneration [70]. RG7800 (RO6885247) is an oral, selective *SMN2* splicing modifier that has passed through a phase 1 clinical trial with promising results such as increasing the SMN protein level up to 2-fold [71]. Hoffmann-La Roche conducted the clinical trial named MOONFISH for testing RG7800, which was terminated due to unexpected toxicology findings in the monkey study (NCT02240355). Unfortunately, the study was put on hold due to nonreversible histological retinal toxicity observed in cynomolgus monkeys [72]. The limitation and potential of RG7800 have led to the discovery of Risdiplam (RG7916). Risdiplam is the splicing modifier of the *SMN2* gene and the first approved oral medication to treat SMA, which was approved by the FDA in August 2020. A Phase 1 study with healthy volunteers resulted in 41% of the maximum increase in *SMN2* mRNA for the highest testing dose [73]. Phase 2 clinical trials are currently active, including 62 participants and 231 patients, which aim to study the safety, tolerability, pharmacokinetics, and pharmacodynamics in infants with type 1 SMA (NCT02913482), type 2 and 3 patients (NCT02908685), respectively. Unlike AO, risdiplam does not require intrathecal injection. The wide range of distribution and effective brain penetration make risdiplam a superior candidate for treating SMA [72].

Although there are approved treatment options, limitations such as high cost and medical morbidity remain. Researchers are focusing on direct treatment methods and aim to maximize the therapeutic effects by combining with other treatment options. The earlier treatment initiation is beneficial for SMA patients. To maximize the therapeutic advantages, fetal gene therapy using mouse model results in increased lifespan in the treatment group compared to the control group. The study aims to measure human SMN gene expression after the intrauterine correction of gene expression in an SMA mouse embryo [74]. The pathogenesis of SMA is complex and could affect more than motor neurons. Altered functions of neuromuscular junctions (NMJs) have been associated with pathological phenotypes of SMA. NMJs in SMA patients show pathological features including immaturity, denervation, and neurofilament accumulation [75]. SMN is crucial for NMJ formation and maturation. Kaiser et al. proposed that the protection and maintenance of NMJs can have therapeutic effects toward treating SMA. The results of administering scAAV9-DOK7 to an intermediate mouse model of SMA demonstrated potential therapeutic effect by improved grip strength, increased weight gain, and extension in survival compared to the untreated group. DOK7, an NMJ organizer, is a novel protective modifier of SMA. Nevertheless, scAAV9-DOK7-treated SMA mice show a significantly increased NMJ endplate area. DOK7 could be targeted in combination with highly productive, SMN-inducing therapies to enhance the integrity of affected NMJs with advantage of prolonging the therapeutic window for SMA treatment [76].

## 4. X-Linked Myotubular Myopathy (XLMTM)

A member of centronuclear myopathy, XLMTM is a rare form of NMD that presents with myopathy, hypotonia [77], respiratory distress [78] and when involving the respiratory muscles is accompanied by high mortality (around 47%) [79]. Mutation in the active site of tyrosine phosphatase myotubularin-encoding gene *(MTM1*) results in pathological features such as a smallness of myofibers, centrally nucleated myofibers mislocation organelles [80], and ultimately the phenotype of muscle fiber disorganization [81]. XLMTM predominantly affects males with 25% of boys dying in their first year [77] and others who survive often requiring extensive supportive care such as a ventilator [79].

### 4.1. AAV-MTM1

Gene therapy aims to correct target muscle weakness through repairing nuclei and mitochondria positioning by delivering the myotubularin-expressing AAV [82]. Canine models showed promising improvement in muscle weakness and respiratory impairment with AAV8-MTM1 [83]. A long-term canine study showed increased body weight and hindlimb length and increased peak inspiratory flow [84]. Nevertheless, some critical differences need to be considered with animal studies. XLMTM in patients often presents with respiratory failure at birth. In contrast, animals such as mice and dogs develop respiratory weakness later in life [83]. Unlike animal models, treatment strategies for human patients should prioritize repairing respiratory functions. Current clinical studies include the phase I/II clinical trial of AT132, which is an AAV8 vector containing a functional copy of the human *MTM1* gene. The study aims to examine the safety and efficacy of AT132 in patients with XLMTM aged less than 5 years with a single dose and followed for five years (NCT03199469). The trial is in process with no published results yet (Table 4).

### 4.2. Antisense Oligonucleotides

Several studies are focused on modulating dynamin 2 (DNM2), which is a protein involved in producing microtubule bundles. The mutated protein has previously been associated with two other hereditary NMDs: Charcot–Marie–Tooth neuropathy and centronuclear myopathy [85]. By increasing the expression levels of DNM2 in mice, researchers observed a phenotype of XLMTM and found the crucial association between the protein and this neuropathy [86]. Then, Cowling et al. concluded that restoration of lifespan, whole-body and muscle strength, and diaphragm function can be achieved by reducing the expression of the DNM2 protein [86]. Studies conducted on centronuclear myopathy knock-in mice also confirmed that DNM2 reduction rescued *DNM2*-related centronuclear myopathy [87]. A preclinical trial aimed to use AOs in mediating the reduction of the DNM2 protein level [88]. Tasfaout et al. found that AO-mediated *DNM2* knockdown could efficiently correct MTM1-induced muscle defects and serve as an attractive therapeutic strategy [88]. Currently, DYN101, an AO therapeutic designed by Dynacure to modulate the expression of DNM2 entered an early-phase trial clinical trial (NCT04033159) on centronuclear myopathy patients. Phase I/II study aims to examine the drug’s safety and efficacy first, a single dose and 4 weeks follow-up assessment followed by a washout period of at least 12 weeks. The second part assesses the multiple-dose treatment of 12 weeks of weekly treatments (NCT04033159). The results of both studies remain to be published.

## 5. Limb-Girdle Muscular Dystrophy Type 2C (LGMD2C)

LGMD is a group of inherited autosomal recessive NMDs characterized by weakness and loss of proximal muscles by a childhood onset. It is one of the most common heterogeneous non-congenital NMDs. The Sarcoglycan-gamma (*SGCG*) gene encodes α-, β-, γ-, and δ-sarcoglycans, which are components of the sarcoglycan complex [89]. The sarcoglycan complex plays a vital role in muscle and non-muscle tissues, especially in stabilizing and protecting sarcolemma against muscle contraction damage, stabilizing the plasma membrane in cardiac and skeletal muscles [90]. Unstable truncated γ-sarcoglycan protein results from a single nucleotide deletion that forms a stop codon and produces an γ-sarcoglycan protein that is merely 35 kDa [89]. There are currently no approved treatments for LGMD2C apart from supportive care. Although clinical trials on exon skipping and AAV gene delivery produced promising results, researchers struggled to identify suitable patients for trials as LGMD2C is often accompanied by other comorbidities, confounding diagnosis.

### 5.1. Multi-Exon Skipping

Exon skipping mediated by AO blocks the pre-mRNA splicing sites to bypass the mutations and modifies the range of exons expressed to mRNAs [91]. In the LGMD2C-associated *SCGC* genotype, a single nucleotide deletion in exon 6 (521ΔT) is the most common mutation, which leads to disruption of the transcript reading frame. The reading frame restoration is achieved by manipulating skipping exons 4–7 and expression of exons 2, 3, and 8 that generate an internally truncated transmembrane protein, which is termed mini-γ [91]. As the AO is designed to skip exons 4–7, it may be assumed that the drug may neutralize any mutation in the *SGCG* that resides in exons 4–7 [91]. By targeting single thymine deletion in exon 6, Wyatt et al. successfully expressed γ-sarcoglycan proteins in patient-derived fibroblasts [91]. Previous studies showed that vivo-PMOs appeared to have better efficacy of skipping and uptake into cells [92]. After administering a multi-exon-skipping cocktail of vivo-PMO oligomers, the reading frame of 521ΔT was effectively restored in myogenically reprogrammed cells from a patient with LGMD2C [92]. Employing the vivo-PMO, Wyatt et al. demonstrated efficient skipping of the targeted exons and functional protein [91]. Unfortunately, targeting exon skipping has yet to reach clinical trials, and unlike in the case of SMA and DMD, there are currently no approved curative gene therapies for LGMD2C. The main approaches toward treating the disease are limited to neutralizing antibodies and immune response [92].

### 5.2. AAV-γ-SGC

Under the phase I clinical trial (Genethon, France, 2011) (Table 5), AAV-1 induced γ-sarcoglycan protein expression with no serious adverse effects (NCT01344798) and moved into a dose-escalation study to investigate its efficiency. A Phase I clinical trial recruited nine wheelchair-dependent patients with homozygous del525T mutation of the γ-SGC gene on chromosome 13 [93]. Assessment tests were conducted every 30 days from 30 days before the treatment until 180 days after the treatment. AAV1.des.hγ-SGC is injected into the non-dominant forearm through the aponeurosis [93]. The results showed no adverse effects except for fever observed in one of the nine patients on day 3 [93]. Israeli et al. studied the vector AAV2/8 in mice and controlled the expression of the *SCGC* cDNA with the muscle-specific promoter to allow for a systemic administration mode while reducing systemic toxicity [94]. Upon validating that the functionality of AAV-8desm-hSGCG is sufficient in correcting the localization of myofibers and reducing dystrophic features, Israeli et al. found that the therapeutic effect is dosage-dependent. The lowest dose only shows less than 5% of γ-sarcoglycan positive fibers, compared to 25% to 75% for intermediate dosage and 75% to 100% for high dosage [91,94]. The study concluded that dosage needs to be considered to achieve effective muscle protection for the treatment.

## 6. Facioscapulohumeral Muscular Dystrophy (FSHD)

Facioscapulohumeral Muscular Dystrophy (FSHD) is one of the most common muscular dystrophies with a prevalence ranging between 2.03 and 6.8 per 100,000 persons [95]. The disease onset typically occurs in the twenties but is also detected at other ages. The muscle weakness deteriorates as the onset persists in a classic pattern: first, the facial and shoulder girdle muscles are affected, then the lower extremities, both distal and proximal. Variations in the age of onset, disease pattern, disease progression, and severity of muscle weakness among FSHD individuals results in disease severity ranging from mildly affected/asymptomatic to wheelchair-bound (20% of FSDH patients) [96,97]. Extramuscular manifestations include hearing loss [98,99] and loss of vision resulting from retinal vascular abnormalities [100,101], exudative retinopathy, and Coat’s syndrome [102].

Autosomal dominant FSHD is caused by the derepression of *DUX4*, a transcriptional regulator, whose target genes are toxic to skeletal muscle [103,104,105]. Unlike DMD, which is caused by mutations in the coding region of a single gene, FSHD requires mixed aberrations of genetic and epigenetic nature to result in *DUX4* derepression and cause clinical symptoms. *DUX4* is located on D4Z4 macrosatellite repeat array on chromosome 4q35 with each D4Z4 repeat (3.3 kb) including a *DUX4* gene, and it is expressed in germline cells but silenced in somatic tissues [103,106]. The silencing can be reversed as a result of DNA hypomethylation and consequent opening of the chromatin structure by the following two mechanisms. In facioscapulohumeral muscular dystrophy type 1 (FSHD1) accounting for 95% of cases, an internal contraction of D4Z4 repeats occurs, leading to a reduced number of repeats, 1 to 10 repeat units, while the unaffected individuals contain 11 to 100 D4Z4 units [106,107]. The contraction results in chromatin relaxation that triggers *DUX4* transcription. Facioscapulohumeral muscular dystrophy type 2 (FSHD2), characterized by a relatively mild phenotype, occurs through a contraction-independent mechanism. About 80% of FSHD2 patients have a mutation in the structural maintenance of chromosomes flexible hinge domain containing 1 gene (*SMCHD1*) on chromosome 18, whose function is to repress *DUX4* expression epigenetically [108]. D4Z4 derepression causing FSHD2 can also result from a mutation in the gene encoding DNA methyltransferase 3B (*DNMT3B*) [109].

The chromatin structure opening in the D4Z4 region alone is not sufficient to induce the *DUX4* expression. The *DUX4* gene lacks a polyadenylation sequence, and *DUX4* mRNA is normally broken down by the cell. The 4qA haplotype contains a polymorphic polyadenylation signal (PAS) distal to the last D4Z4 repeat that can be added to nascent *DUX4* transcripts, stabilizing them and leading to the translation of DUX4 proteins. [110,111,112] DUX4 is a rare protein; after all, there must be a downstream cascade that can magnify its effect. Paired-like homeodomain transcription factor 1 (*PITX1*) and p53 are the factors that are up-regulated in FSHD patients. The DUX4 protein activates the *PIXT1* promoter followed by activation of p53, PITX1 diffusion, and deregulation cascade was speculated to cause widespread muscle defects [113,114,115].

Despite the progress made in understanding the mechanism of *DUX4* derepression, the details on transcriptional factors and gene activation mechanisms remain largely unknown. Until recently, several factors involved in the signaling pathway have been reported intermittently. It was recently reported that the p38 inhibitor suppresses *DUX4* mRNA expression in FSHD1 and 2 derived myoblast and differentiation myocytes [116]. p38α/β is identified to play a critical role in the downstream transcription pathway and thus result in an aberrant activation of *DUX4*. Successively, the p38α/β inhibitor showed robust a downregulation of *DUX4* expression in patient-derived FSHD1 and FSHD2 cell lines [117]. Hyaluronic acid has been reported as a signaling molecule in the mechanism of DUX4-induced molecular pathologies, which is indicative of its potential to be a therapeutic target [118]. Several G-quadruplex-forming sequences were discovered in the *DUX4* locus, and berberine, a G-quadruplexes ligand, later proved to suppress *DUX4* expression in patient-derived myoblasts and increase muscle strength in mouse models [119]. Although the picture is unlikely to be complete and a greater discovery of *DUX4* expression factors are needed so that researchers could establish a more comprehensive pathogenic mechanism, these findings can still serve as novel therapeutic targets to treat FSHD.

Currently, pharmacological disease-modifying treatments in FSHD are still in the phase of trial research. Putative molecules or drugs that could either reduce the loss of muscle mass or help muscle strength restoration includes prednisone with anti-inflammatory effects; β2-adrenergic agonists with anabolic effects; MYO-029, a neutralizing antibody to myostatin; diltiazem, a calcium channel blocker, antioxidants vitamin C, vitamin E, zinc gluconate, and selenomethionine that might help reduce oxidative insults; and finally testosterone. However, these trials turned out to have limited significant improvements in terms of muscle strength or functions [120].

### 6.1. RNA Interference through AAV Vector

One of the first RNA interference (RNAi) strategies applied in FSHD was tested in the 2010s. In 2010 and 2011, Wallace et al. constructed U6-promoter-driven FSHD region gene 1-targeted microRNA (U6-miFRG1). The microRNA was later cloned into AAV.CMV.hrGFP to construct AAV.miFRG1. It was injected into mice expressing toxic levels of human FRG1 (FRG1−high mice). Using the same strategy but with *DUX4* as the target of gene silencing is demonstrated, and AAV.miDUX4 was cloned. Improvements in the histologic evidence of muscle mass and functional muscle abnormalities were observed [121,122]. Likewise, another construct rAAV6-sh1FRG1 systemically delivered to FRG1 mice resulted in a significant rescue of the disease with histological, molecular, and muscle function evidence. Thus, rAAV6-sh1FRG1 is a potential, dose-dependent, long-term RNAi therapy for FSHD [123]. Performing a safety profile in preclinical stages is crucial for further demonstration in human trials, especially in gene therapies such as the endogenous RNAi pathway, as they were cautioned that the saturated synthesis of natural miRNA induces toxicity and unexpected gene silencing occurs in off-target genes. rAAV6-sh1FRG1 is concluded to be safe in mice, as no toxicity events were observed in heart and liver tropism, there was a normal level of inflammation, and there was no interference in the natural miRNA synthesis [123]. However, scAAVmi1155 (a miDUX4) did not deliver a safety profile, and scAAVmi405 only appears safe at low doses [124].

### 6.2. Antisense Oligonucleotides (AOs) and Phosphorodiamidate Morpholino Oligomers (PMOs)

#### 6.2.1. Modulation of PITX1

Phosphorodiamidate morpholino oligomers (PMOs) are short chains of DNA sequence that have been used to target transcriptional starting sites and regulate gene expression. A modified PMO, Octaguanidinium dendrimer-conjugated morpholino (vivo-morpholino)—in which morpholino is used to block the PITX1 mRNA initiation transcript, and octaguanidium dendrimer conjugation helps morpholino penetrate the cell—is demonstrated on PITX1 transgenic mice. The vivo-morpholino performed positive results that could suppress PITX4 protein expression, reduce muscle atrophy, and improve grip strength without major adverse events in animal models [125].

#### 6.2.2. Pre-mRNA Level

Polyadenylation is an essential step that stabilizes eukaryotic mRNAs; thus, it is also a potential therapeutic target of FSHD. Phosphorodiamidate morpholino oligonucleotide FM10, which targets the polyadenylation signal, reduced *DUX4* protein levels without significant cell toxicity and overt off-target effects in both FSHD myogenic cells and human muscle xenografts [126]. PMO-PAS (GGGCATTTTAATATATCTCTGAACT) targets 3′ end polyA sites 766 bp downstream of the stop codon and PMO-CS3 (TATAGGATCCACAGGGAGGAGGCA TTTTAA) targets cleavage sites of pre-mRNA. The two PMOs down-regulate DUX4 expressions, indicating an eligible strategy in FSHD treatment [127]. Antisense 2′-O-methoxyethyl (2′-MOE) gapmers induce RNase H activation and thus the knockdown *DUX4* mRNA expression by reducing transcript levels immortalized patient-derived muscle cells and in the FLExDUX4 FSHD mouse model [128].

## 7. Discussion

AAVs are recognized as the most common tool for the gene therapy-based treatment of NMDs. AAVs are known for recognizing and targeting certain cell types and then carrying its genetic material into the cell to further modify the genetic sequence of the host cell. The application AAV is challenged in DMD due to the gene sequence being too large (14 kb) for a small vector (packaging capacity of AAV is around 4.7 kb) [129]. The researchers need to test for the small enough gene to fit in the vector while still maintaining the therapeutic effect. Preclinical trials in animals had successfully been conducted with promising results. As of March 2020, the clinical trial for DMD patients using IV delivering micro-dystrophin is in Phase 1/2a with micro-dystrophin expression at the correct location (NCT03375164). Future goals will be recruiting more patients to test for safety and efficacy with long-term effects, since the sample size of clinical trials mentioned previously was too small to generalize.

One of the most challenging parts of AAV-based therapy is adjusting the dosage, as high levels of AAV may lead to toxicity. Indeed, AAV trials have been reported with dose-dependent results. Another challenge is the difficulty of transitioning from animal studies to human studies due to animals’ different physiology symptoms. For example, although AAV studies in canine models with XLMTM showed promising results such as increased body weight and limb length, the applicability to humans may be challenged by insufficient focus on investigating the respiratory failure, since it is the earliest and most fatal symptom found in humans, but not in canines. Just as in the case of DMD, AAV-based clinical trials for LGMD2C and XLMTM are in their early stages. Although it is difficult to overcome all the challenges of developing safe and efficient AAV treatment, advanced technology and increased research have shown helpful prospects. Approved AAV therapy includes voretigene neparvovec (Luxturna) using AAV2-hRPE65v2 for treating retinal dystrophy. It is the first FDA-approved gene therapy drug for inherited disease, which has been approved in December 2017. Another FDA-approved AAV-based gene therapy is onasemnogene abeparvovec (Zolgensma), which is an AAV9-based method to treat SMA that was approved in May 2019. There comes another challenge after the successful development and approval of the treatment. Financing in capital marketing will be a challenge due to the high cost and risks during development. Many of the patients with the disease cannot afford the treatment. This also creates a challenge to the insurance system.

Approaches based on modulating different stages of the gene expression pathway, including exon-skipping, mutation suppression, and AAV-mediated gene transfer have been shown to ameliorate the phenotype generated by mutations in the corresponding genes. Nevertheless, none of the methods result in the complete cure of the disease. Turning to gene-editing strategies such as CRISPR/Cas9 is of great potential to achieve permanent cure of the genetic intractable diseases [130,131,132,133]. Although ASOs achieved a therapeutic expression of dystrophin protein in multiple clinical trials, their therapeutic benefit is limited by their short half-lives. In contrast, genome editing with the CRISPR/Cas9 technology promises permanent genomic corrections and a life-long cure [134,135]. According to the Leiden DMD database, out of roughly 5000 identified pathogenic mutations, there are 65% deletions, 27% point modifications, and 8% are duplications [136,137,138]. However, the most well-known ASOs are designed to skip exon 51 harboring the largest proportion of mutations, even so, it is estimated to be applicable to only ≈10% of DMD patients [134,135]. The vastly uncharted bulk of mutations demands a more pragmatic and versatile gene therapy platform. Herein, researchers turned to CRISPR/Cas9 to broaden the targetable mutations and cure previously neglected variants [139,140,141]. With the common goal of restoring the reading frame and functional dystrophin expression, the research community evaluated multiple designs to implement a range of genome editing strategies that we will delineate from smaller genetic manipulations to larger multi-exon deletions and duplication corrections.

In the absence of a donor template, the Cas9-cleaved DNA is repaired via an error-prone repair mechanism, non-homologous end joining (NHEJ), whereby one or more nucleotides are randomly integrated or deleted to create the indels. Indel formation can be exploited to directly disrupt the splice acceptor sites [142], mutate downstream nonsense mutations to read through premature stop codons [143], or by integrating indels in surrounding exons, enabling smooth read-through between exons [144]. Nevertheless, indel methods face the challenges of reproducibility and consistency to address each patient [145]. For mutated transcripts that require the excision of exons to produce in-frame mRNA, researchers can also delete whole exons with CRISPR/Cas9 [133,144,146,147]. Deleting an exon requires paired gRNAs flanking the mutated region to induce exon deletion [147,148] and subsequent fusion of the targeted exons. By using these methods, the transcript’s critical domains could be restored, such as a spectrin-like repeat of the dystrophin rod domain [148] and central rod-domain and correct spectrin-like repeat [149]. Such domain restoration is not readily obtained by ASO-induced exon skipping. As the addition of gRNA comes with an increased risk of off-target, researchers attempted to excise exons with a single gRNA [150,151], which prompted the investigation of a possibility to excise larger regions that span multiple exons. By doing so, researchers could cover major mutational hotspots and target a broader spectrum of patients at once. Among the large deletions and large duplications, 66% and 15%, respectively, are located between exons 45 and 55, constituting a so-called major hotspot region [26]. Attempting to address multiple exon skipping, researchers envisioned the use of numerous gRNAs or Cas enzymes expressed at once to target multiple loci, which was termed multiplexed CRISPR [132,152]. The unique multiplex gene-editing system facilitates the generation of a single large deletion that can correct up to 62% of DMD mutations [132,153]. Maggio et al. boldened the concept and studied CRISPR/Cas9 multiplexes’ potential in unselected populations of DMD. Multiplexed DMD editing triggered short- and long-range intragenic DMD excisions. By allowing the synchronous and stoichiometric expression of the various gene-editing components, Maggio et al. demonstrate a correction of over 10% of target alleles in the unselected population [152]. Using paired gRNAs, Brescia et al. corrected up to 42% of defective DMD alleles through the targeted removal of the major mutational hotspot (>500 kb) [143]. Challenging the largest deletion accomplished to date, Young et al. induced the deletion of exon 45–55 (725 kb) with a single gRNA [145,153]. The technology to excise large frame-shift mutations is further applied to exonic duplications, which is a distinct group that has been generally neglected by therapeutic approaches. Exonic duplications account for 10–15% of all mutations in DMD. For patients carrying disease-causing duplications, Wojtal et al. hypothesize that a single gRNA designed over a tandem duplicated region will cut twice, leading to the removal of duplication [154]. The authors removed exon duplications (exon 18–30, 2) and achieved full-length dystrophin production targeting duplication junctions with a single gRNA [154,155].

With the advent of CRISPR/Cas9 and successful preclinical studies, researchers aim to expand the coverage of treatable diseases. Apart from DMD, some interesting developments are happening in targeting SMA and LGMD. For SMA, researchers turn to the genetic conversion of SMN2 to SMN1 to rescue full-length functional SMN expression. For this purpose, an artificial splicing factor (CASFx) was engineered for CRISPR-induced alternative splicing and Cas analog Cpf1 directed HDR to correcting *SMN2* exon 7 [156,157]. CASFx modulates alternative splicing and allows simultaneous exon inclusion and exclusion by differential positioning of the factor [156,158]. When tackling LGMD, researchers expanded the target group of LGMD2C/SGCG [92] and explored the mutations in LGMD2A/CAPN3 [159], LGMD2G/TCAP [160], and LGMD2B/DYSF [161]. The versatility of the CRISPR platform is again demonstrated as authors employed microhomology-mediated end joining for microduplications, HDR for nonsense mutations, and gene knock-in [159,160,161]. The booming field of genome editing has flourished, and researchers have now gained the necessary tools in tackling previously intractable diseases. Exciting research with novel strategic gene-editing methods is on the surge, and the research community is anticipating a wave of unprecedented ideas in future studies.

## 8. Conclusions

Readily available in a standardized way, next-generation sequencing serves as the first-line diagnosis of NMDs for which a wide range of genetic defects present with similar clinical manifestations. Gaining momentum and standardization in clinical practice, molecular diagnosis allows for gene-targeting and editing therapeutics to thrive. Genome sequencing has revolutionized how researchers understand genotype–phenotype associations and the interplay between the nature of the mutation with the severity of the clinical phenotype. These advancements provide promising insights into the eventual translation of gene therapy into clinical therapeutic reality for diseases that to this date remained intractable.

## Figures and Tables

**Table 1:** Clinical trials of Duchenne muscular dystrophy gene therapy.

Class	Phase	Enrolled #	Therapeutics	Route	End Date	Start Date	Trial ID
Exon Skipping	1/2	7	Eteplirsen (AVI-4658/Exondys-51)	IM	December 2008	October 2007	NCT00159250
	1/2	19	Eteplirsen (AVI-4658/Exondys-51)	IV	December 2010	January 2009	NCT00844597
	2	12	Eteplirsen(AVI-4658/Exondys-51)	IV	June 2012	July 2011	NCT01396239
	2	6	Casimersen (skip exon45), Eteplirsen (skip exon51), Golodirsen (skip exon53)	IV	September 2021	February 2020	NCT04179409
	2	53	Drisapersen (GSK2402968)	SC	September 2012	September 2010	NCT01153932
	2	51	Drisapersen (GSK2402968)	SC	May 2013	October 2011	NCT01462292
	3	186	Drisapersen (GSK2402968)	SC	June 2013	December 2010	NCT01254019
AAV gene transfer	1	2	SRP-9001 (rAAVrh74.MHCK.microdystrophin)	IM	September 2017	March 2015	NCT02376816
	1	6	d3990 (rAAV2.5-CMV-minidystrophin)	IM	March 2009	March 2006	NCT00428935
	1	15	d3990 (rAAV2.5-CMV-minidystrophin)	IV	August 2021	January 2018	NCT03362502
	1//2	4	PF-06939926 (AAV9- muscle specific promoter- minidystrophin)	IV	March 2020	January 2018	NCT03375164
	1//2	6	SRP-9001 (rAAVrh74.MCK.microDystrophin)	IV	November 2020	November 2017	NCT03333590
	1//2	3	rAAVrh74.MCK.GALGT2	IM	November 2017	January 2015	NCT02354781
	1//2	3	AAV1-F344 (rAAV1.CMV.huFollistin344)	IV	January 2023	January 2020	NCT04240314
	1//2	16	scAAV9.U7.ACCA (skip exon2)	IV	March 2023	December 2017	NCT03368742
	2	41	SGT-001 (AAV9- muscle specific promoter- microdystrophin)	IM	October 2022	December 2018	NCT03769116
	3	99	SRP-9001 (rAAVrh74.MHCK.microdystrophin)	IV	June 2022	July 2020	NCT03368742
	1	2	PF-06939926 (AAV9- muscle specific promoter- minidystrophin)	IM	September 2017	March 2015	NCT02376816
	1	6	SRP-9001 (rAAVrh74.MHCK.microDystrophin)	IM	March 2009	March 2006	NCT00428935
	1	15	d3990 (rAAV2.5-CMV-minidystrophin)	IV	August 2021	January 2018	NCT03362502
	1//2	4	d3990 (rAAV2.5-CMV-minidystrophin)	IV	March 2020	January 2018	NCT03375164
	1//2	6	PF-06939926 (AAV9- muscle specific promoter- minidystrophin)	IV	November 2020	November 2017	NCT03333590
Nonsense Suppression	2	6	Ataluren (PTC124)	PO	March 2010	January 2010	NCT01009294
	2	14	Ataluren (PTC124)	PO	February 2018	June 2016	NCT02819557
	2	36	Ataluren (PTC124)	PO	May 2010	July 2008	NCT00759876
	3	95	Ataluren (PTC124)	PO	January 2018	May 2012	NCT01557400
	2	173	Ataluren (PTC124)	PO	May 2010	January 2009	NCT00847379
	2	174	Ataluren (PTC124)	PO	December 2009	February 2008	NCT00592553
	2	21	Arbekacin sulfate (NPC-14)	PO	October 2015	August 2013	NCT01918384
	2	38	Ataluren (PTC124)	PO	May 2007	December 2005	NCT00264888
	3	230	Ataluren (PTC124)	PO	August 2015	March 2013	NCT01826487
	2	6	Ataluren (PTC124)	PO	March 2010	January 2010	NCT01009294
	2	14	Ataluren (PTC124)	PO	February 2018	June 2016	NCT02819557
	2	36	Ataluren (PTC124)	PO	May 2010	July 2008	NCT00759876

# Number, IM intramuscular; IV intravenous; PO oral; SC subcutaneous.

**Table 2:** Clinical trials for Becker muscular dystrophy.

Class	Phase	Enrolled #	Therapeutics	Route	End Date	Start Date	Trial ID
AAV gene transfer	1	15	AAV1-F344 (rAAV1.CMV.huFollistin344)	IM	October 2017	January 2012	NCT01519349
Nonsense suppression	2	6	Ataluren (PTC124)	PO	March 2010	January 2010	NCT01009294
	2	173	Ataluren (PTC124)	PO	May 2010	January 2010	NCT00847379

# Number, IM intramuscular; PO oral.

**Table 3:** Clinical trials for spinal muscular atrophy gene therapy.

Class	Phase	Enrolled #	Therapeutics	Route	End Date	Start Date	Trial ID
Alternative splicing	1	33	Evrysdi (Risdiplam)	PO	August 2016	January 2016	NCT02633709
	1	33	Evrysdi (Risdiplam)	PO	August 2016	January 2016	NCT02633709
	2	25	Evrysdi (Risdiplam)	PO	June 2021	August 2019	NCT03779334
	2/3	62	Evrysdi (Risdiplam)	PO	November 2023	December 2016	NCT02913482
	1/2	34	Spinraza (Nusinersen)	IT	January 2015	October 2012	NCT01703988
	3	122	Spinraza (Nusinersen)	IT	August 2023	November 2015	NCT02193074
	3	292	Spinraza (Nusinersen)	IT	November 2016	August 2014	NCT02594124
	1/2	40	Branaplam (LMI070; NVS-SM1)	PO	July 2020	April, 2015	NCT02268552
	1	9	RG7800 (RO6885247)	PO	July 2015	November 2014	NCT02240355
AAV Gene Therapy	3	2	Zolgensma (Onasemnogene Abeparvovec-xioi; AVXS-101)	IV	June 2021	May 2019	NCT03837184
	3	22	Zolgensma (Onasemnogene Abeparvovec-xioi; AVXS-101)	IV	November 2019	October 2017	NCT03306277
	3	30	Zolgensma (Onasemnogene Abeparvovec-xioi; AVXS-101)	IV	June 2021	April, 2018	NCT03505099
	3	33	Zolgensma (Onasemnogene Abeparvovec-xioi; AVXS-101)	IV	September 2020	August 2018	NCT03461289
	3	15	Zolgensma (Onasemnogene Abeparvovec-xioi; AVXS-101)	CVC	December 2017	May 2014	NCT02122952
	4	308	Zolgensma (Onasemnogene Abeparvovec-xioi; AVXS-101)	IV	December 2035	February 2020	NCT04042025

# Number, PO oral; IT intrathecal; IV intravenous; CVC central venous catheter.

**Table 4:** Clinical trials for X-linked myotubular myopathy (XLMTM).

Class	Phase	Enrolled #	Therapeutics	Route	End Date	Start Date	Trial ID
AAV Gene Transfer	1/2	24	AT132 (AAV8-DES-hMTM1)	IV	March 2020	June 2017	NCT03199469
Antisense Oligonucleotide	1/2	18	DYN101 (IONIS-DNM2–2.5Rx)	IV	April 2020	January 2020	NCT04033159

# Number, IV intravenous.

**Table 5:** Clinical trials for limb girdle muscular dystrophy Limb-girdle muscular dystrophy Type 2C (LGMD2C) gene therapy.

Class	Phase	Enrolled #	Therapeutics	Route	End Date	Start Date	Trial ID
AAV gene transfer	1	9	AAV1-γ-sarcoglycan	IM	June 2010	November 2006	NCT01344798

# Number, IM intramuscular.
